# Identifying central symptom clusters and correlates among post-COVID-19 pulmonary fibrosis patients: a network analysis

**DOI:** 10.3389/fmed.2025.1538708

**Published:** 2025-05-21

**Authors:** Zhen Yang, Zhiqin Xie, Zequan Wang, Linxia Yi, Shihan Chen, Yunyu Du, Xuemei Tao, Chao Xie, Li Zhou, Min Zhang, Chaozhu He

**Affiliations:** ^1^Department of nursing, The First Affiliated Hospital, Jiangxi Medical College, Nanchang University, Nanchang, Jiangxi, China; ^2^School of Nursing, Jiangxi Medical College, Nanchang University, Nanchang, Jiangxi, China; ^3^Jiangxi Medical Center for Critical Public Health Events, The First Affiliated Hospital, Jiangxi Medical College, Nanchang University, Nanchang, Jiangxi, China; ^4^Department of Thoracic Surgery, The First Affiliated Hospital, Jiangxi Medical College, Nanchang University, Nanchang, Jiangxi, China

**Keywords:** COVID-19, pulmonary fibrosis, lung diseases, interstitial, syndrome, social network analysis

## Abstract

**Background:**

Previous studies have analyzed symptom clusters in patients with coronavirus disease 2019 (COVID-19); however, evidence regarding the core symptom clusters and their influencing factors in patients with post-COVID-19 pulmonary fibrosis (PCPF) remains unclear, affecting the precision of symptom interventions.

**Objectives:**

This study aimed to identify the symptom clusters and core symptom clusters in patients with PCPF. Demographic and disease-related factors associated with these symptom clusters were also analyzed.

**Methods:**

A total of 350 patients with PCPF were recruited from China between January 2023 and April 2024. A self-reported symptom assessment scale was used for this survey. Principal component analysis was used to identify symptom clusters. Network analysis was used to describe the relationships between the symptoms and symptom clusters. Multiple linear models were used to analyze the factors affecting the total symptom severity and each symptom cluster.

**Results:**

Six symptom clusters were identified: Upper Respiratory Tract Symptom Cluster (USC), Lower Respiratory Tract Symptom Cluster (LSC), Somatic Symptom Cluster (SSC), Muscular and Joint Symptom Cluster (MSC), Neurological and Psychological Symptom Cluster (NSC), and Digestive Symptom Cluster (DSC). Fatigue was identified as the core and bridge symptom in the symptom network, whereas the upper respiratory symptom cluster was identified as the core and bridge symptom cluster. Gender, age, educational level, smoking history, and primary caregiver were associated with the scores of the six symptom clusters.

**Conclusion:**

Our study suggests that there is a need to evaluate symptom clusters for the improvement of symptom management among PCPF. Specifically, the assessment and treatment of upper respiratory and fatigue symptoms as core targets of PCPF care is critical for the development of accurate and efficient symptom management strategies.

## 1 Introduction

As of April 28, 2024, the World Health Organization reported 775,379,864 confirmed cases of the coronavirus disease 2019 (COVID-19) and 7,047,396 deaths due to respiratory failure and other major organ damage (1). With the evolution of the SARS-CoV-2 virus, the availability of multiple vaccines, and improvements in clinical management, mortality rates have steadily declined (2–4), but the incidence rate remains high. As of early May 2024, China has reported 1,400 new cases per week (1). Most patients with COVID-19 experience mild symptoms and recover within 1–2 weeks (5). However, 6%−68% of COVID-19 survivors have persistent symptoms, a condition known as “Long COVID” (6, 7). COVID-19 pneumonia can lead to fibrotic lung damage, known as post-COVID-19 pulmonary fibrosis (PCPF), which is a common complication in patients with Long COVID (8). Among all hospitalized COVID-19 survivors, 45% to 54% develop pulmonary fibrosis (9). One year later, one-third of all patients with moderate COVID-19 exhibited fibrotic changes that severely impaired lung diffusion capacity (10). There have also been reports of pulmonary fibrosis following an asymptomatic COVID-19 infection. Patients with pulmonary fibrosis have a lower quality of life and require additional medical care (11, 12). The high prevalence of pulmonary fibrosis has become a serious global issue (13). However, the underlying mechanisms remain unclear, and treatment methods are yet to be developed (10).

Post-COVID-19 is a complex and heterogeneous disease that may involve multiple organ systems and various symptoms, including fatigue, headache, shortness of breath, loss of smell or taste, and diarrhea (14). The mechanisms behind Long COVID are complex and uncertain (15–17). Some studies suggest that certain symptoms of Long COVID may be related to the persistence of the virus in related tissues, including the intestinal mucosa or epithelium, olfactory nerve epithelium, taste buds, and blood (18–21). Researchers have described more than 100 post-COVID-19 symptoms, which show some commonalities and significant heterogeneity across different studies (22–24). This vast number of symptoms highlights the challenge of organizing them into meaningful patterns that are useful to clinicians.

Identifying symptom clusters is a classic approach to reducing the dimensionality of symptoms and simplifying the complex relationships between symptoms in clinical practice (25). One symptom within a cluster may affect others (7). According to Kim et al., a “symptom cluster” represents two or more symptoms that occur simultaneously and may or may not have the same etiology (26). Through exhaustive searches, we found 13 studies that identified symptom clusters post-COVID infection. For instance, the Global Burden of Disease Long COVID Collaborators identified three symptom clusters based on the frequency reported in published studies: persistent fatigue-muscle pain-emotional fluctuation, cognitive attention disorder, and persistent respiratory symptoms (27). Paul et al. identified three post-COVID symptom clusters using a similar approach, including cardiopulmonary, inflammatory, and neurological symptoms (28). Larson et al. identified five symptom clusters using exploratory factor analysis, including respiratory or respiratory-muscle, general viral, olfactory/taste, cognitive/cardiac, and mental symptoms (29). Although the above studies have investigated symptom clusters post-COVID infection, the means of identifying symptom clusters in these studies were mainly subjective classification (30), co-occurrence networks (31), cluster analysis (32–37), exploratory factor analysis (38, 39), and literature review (27, 28). However, these methods are unable to determine the interactions between symptoms and symptom clusters. In addition, previous studies have found that differences in age (29–37, 39), disease stage (30, 31, 33, 36, 40), and occupation (40) of the study population can lead to heterogeneity of results. Notably, patients with PCPF are a group of patients with Long COVID with a heavier symptom burden, no studies have explored the symptom clusters in patients with PCPF, and there is an urgent need to identify the symptom clusters of this group of patients.

Network analysis can comprehensively evaluate the relationships between symptoms, visualize the complex relationships between symptoms, and assess the network structure between variables (41), thus serving as a novel approach for identifying core symptoms and symptom clusters. Identifying core symptoms provides healthcare providers with a broad perspective for developing precise intervention strategies.

However, to our knowledge, no study has used network analysis to identify symptom clusters in patients with PCPF, and the most specific core symptom clusters in patients with PCPF remain unclear, which hinders healthcare providers and researchers from fully understanding and developing targeted interventions. Therefore, this study identifies and understands core symptoms and symptom clusters through network analysis, which provides a basis for clinical healthcare professionals to develop accurate and efficient management plans, and helps to improve the efficiency and accuracy of symptom management interventions.

Previous studies have indicated associations between sociodemographic information (gender and age) and symptom clusters (38). However, the relationship between symptom clusters and demographic factors in patients with PCPF has not been established. Therefore, there is a lack of understanding of the factors that influence symptom clusters. To address the current knowledge gap, our study aimed to answer three key research questions: (1) How many symptom clusters exist in patients with PCPF? (2) Which is the most core symptom cluster? (3) Which demographic and health-related factors are associated with these symptom clusters?

## 2 Methods

### 2.1 Study design and setting

We conducted a cross-sectional study using convenience sampling between January 2023 and April 2024. We collected data from 350 patients with PCPF from the Respiratory and Infectious Diseases Department of the First Affiliated Hospital of Nanchang University, Jiangxi Province, China. This hospital is one of the largest in Eastern China and is recognized as a unit of the “Respiratory Disease Difficult and Complicated Disease Diagnosis and Treatment Capacity Improvement Project,” a national major epidemic prevention and treatment base, a national, regional medical center, and an affiliated unit of the Jiangxi Province Major Public Health Event Medical Center. Our hospital has 6,100 beds, with 850 beds in the respiratory and infectious diseases department. The patients mainly came from the Eastern China region, and in 2023, these two departments had a combined annual outpatient volume of 220,000 visits.

### 2.2 Study population

The eligibility criteria included participants: (1) aged ≥ 18 years; (2) diagnosed with PCPF (42); (3) willing to provide written informed consent; (4) having clear normal communication awareness or ability. Participants were excluded if they (1) were diagnosed with mental illness or cognitive impairment or (2) had severe complications, such as heart, lung, or kidney failure, and various malignant tumors.

### 2.3 Sample size

According to the guidelines for calculating the sample size for network analysis (43), the minimum required sample size is P(P-1)/2, where P represents the nodes in the network, that is, the items of the symptom assessment scale. In this study, a symptom assessment scale with 19 items was used, resulting in a minimum required sample size of 171. To ensure the accuracy of our results and the representativeness of the sample, the study ultimately enrolled 350 eligible participants.

### 2.4 Measures

#### 2.4.1 General data survey questionnaire

We designed a structured questionnaire that included demographic data (age, gender, place of residence, educational level, employment status, and marital status) and disease-related information (primary caregiver, smoking history, and comorbidities).

### 2.5 Reported symptoms

Before conducting this study, we reviewed previous research and summarized the assessment tools for symptom clusters in patients with COVID-19. We found that the identification of symptom clusters in previous studies relied mainly on symptom severity (33, 39, 40, 44) and the occurrence or frequency of symptoms (31, 32, 35, 37, 38). Only one study considered the degree of life distress caused by symptoms (34). Therefore, it is necessary to use an assessment tool that comprehensively evaluates symptom occurrence, severity, and distress to identify symptom clusters in patients with PCPF. Our team developed a new measurement method based on four symptom checklists: the Chinese version of the Memory Symptom Assessment Scale (45), the Self-Reported Symptom Questionnaire (46), the Long COVID Self-Reported Symptom Assessment Tool (47), and the inFLUenza Patient-Reported Outcome Plus (FLU-PRO Plus) (48).

We selected 51 high-incidence symptoms using the aforementioned assessment tools. After item and exploratory factor analyses, we finalized 19 highly prevalent symptoms to create a symptom self-assessment scale for patients with PCPF. The scale consists of two parts: the first part includes 17 items that assess symptom occurrence, frequency, severity, and distress, and the second part includes two items that assess symptom occurrence, severity, and distress. If the symptoms described in the item did not occur, it was scored as 0. If it did occur, a Likert 4-point scale was used to measure symptom frequency (1 = rarely, 2 = sometimes, 3 = often, 4 = almost always); severity was measured using a Likert 4-point scale (1 = mild, 2 = moderate, 3 = severe, 4 = very severe), and distress was measured using a Likert 5-point scale (0 = not at all, 1 = a little, 2 = somewhat, 3 = quite a bit, 4 = very much). Higher scores indicated more severe symptoms. The symptom score for each item was the average score of multiple dimensions, and the overall score of the symptom scale was the average score of the 19 items. The symptom self-assessment scale for patients with PCPF demonstrated good reliability and validity with a Cronbach's α coefficient of 0.836.

### 2.6 Data collection

This study was approved by the Ethics Review Committee of the First Affiliated Hospital of Nanchang University, Nanchang City, Jiangxi Province, China (Approval No. 2022-127; Approval Date: April 7, 2022). Owing to the high response rate of paper questionnaires, this study used paper questionnaires to collect data. Six members of the research team (nursing graduate students) received standardized training to collect general demographic data and disease-related information. After obtaining consent from the hospital and department, the patients were recruited through the hospital management system. Disease diagnosis was provided by physicians. Researchers A, B, and C first collected patients' sociodemographic and disease-related information from the medical record system and then distributed the paper-based version of the symptom self-assessment scale to the participants in the wards. The questionnaire was anonymous, and no identifiable information, such as names or patient admission numbers, was recorded. If the participants had any doubts about the questionnaire, they could ask the researchers. Upon completion, researchers A, B, and C immediately reviewed the questionnaires to ensure that no items were missing. If the participants were unwilling to complete any missing items, they were not required to continue. After completing the survey, each participant received a small appreciation token. Researchers D and E reviewed the completed questionnaires and entered them into a computer. All data were stored on a password-protected computer and locked in a cabinet. These data were managed by researcher F. Of the 368 questionnaires distributed, 12 were invalid owing to patterned responses (for example, the same answer for 19 consecutive questions), and six were invalid owing to missing data. A total of 350 valid questionnaires were obtained, with an effective response rate of 95.1%.

### 2.7 Data analysis

Data analysis was performed using Statistical Package for Social Sciences software (version 22) for Windows (IBM, Armonk, NY, USA). We used frequencies, percentages, means, and the Depression Self-Rating Scale to describe the demographic variables and symptom severity.

To detect symptom clustering among the 19 PCPF-related symptoms, principal component analysis (PCA) was used, and the dimensions of the symptoms (severity scores) were determined using the Kaiser-Meyer-Olkin test to assess the suitability of our data for factor analysis. An orthogonal transformation (varimax rotation) was applied to PCA. Factors with eigenvalues >1.0 were included. The number of factors was confirmed using Horn's parallel analysis. Symptoms with factor loadings >0.45 were included in the clusters (49). Cronbach's alpha coefficient was used to evaluate the internal consistency and reliability of the derived factors. Discussions among research team members ensured the clinical relevance of the derived symptom clusters.

An association network was established using the R package “qgraph” to describe the relationships between the symptoms and clusters. In the symptom network, Spearman correlations were used to estimate the relationships between symptom pairs (average scores of each symptom dimension) and symptom clusters (average scores of symptoms within the cluster). Edges represented conditional independence relationships between nodes. The thickness of the edges indicated the strength of the association. To reduce false positives, we used the Least Absolute Shrinkage and Selection Operator to remove small edges and applied the Extended Bayesian Information Criterion with an adjusted parameter γ for an optimal network fit (43, 50). Due to the use of orthogonal transformation in PCA, the influence of individual items was minimized, so all symptom clusters and individual symptoms were included in the network analysis to detect centrality indices (49).

We used three centrality indices, strength, closeness, and betweenness, to identify the most central symptoms and symptom clusters. Strength indicates the absolute sum of the correlation coefficients for edges connected to a node, implying the likelihood of co-occurrence of a symptom with others. Closeness indicates the distance of a node from all other connected nodes, with shorter weighted paths representing higher closeness. Betweenness indicates the number of times a node is placed on the shortest path between two nodes. Symptoms acting as bridges between pairs of symptoms had a high betweenness centrality. Symptoms with the highest centrality coefficients were identified as core symptoms and symptom clusters. By identifying and intervening on a core symptom or cluster of symptoms, the “targeting” effect of other symptoms associated with the symptom can be lost, and the intervention propagates to nodes around the core symptom or cluster of symptoms, thereby alleviating other symptoms and disrupting the linkages between clusters of symptoms to improve the patient's quality of life and further enhance the effectiveness of symptom management.

This study employed exploratory multivariate linear models to investigate the factors associated with overall symptom severity and the severity of six symptom clusters in patients with PCPF. The model included the following demographic and clinical variables: gender (female = 1, male = 0), age, education level (primary school or below = 1, others = 0), residence (rural = 1, urban = 0), smoking history (no = 1, yes = 0), employment status (others = 1, employed = 0), marital status (others = 1, married = 0), primary caregiver (others = 1, self = 0), and comorbidities (yes = 1, no = 0). In all analyses, a two-tailed *P*-value < 0.05 indicated statistical significance.

## 3 Results

### 3.1 Participant characteristics

In total, 350 patients with PCPF were included in this study. The general patient characteristics are presented in Table 1. Most participants were males (60.00%), married (93.43%), and urban residents (67.14%). The mean age was 65.18 years. Among them, 29.71% had an education level of primary school or below, 12.29% were employed, and 8.00% had themselves as their primary caregivers after becoming ill. Additionally, 26.57% had a smoking history, and 61.43% had comorbidities.

**Table 1:** Participant characteristics (*N* = 350).

**Characteristics**	***n* (%), Mean (SD)**
Age	65.18 (15.59)
**Gender**	
Male	210 (60.00)
Female	140 (40.00)
**Residence**	
Urban	235 (67.14)
Rural	115 (32.86)
**Employment**	
Employed	43 (12.29)
Otherwise	307 (87.71)
**Primary caregiver**	
Myself	28 (8.00)
Family members (spouse, parents, kids, or other relatives)	317 (90.57)
Otherwise	5 (1.43)
**Education level**	
Primary school or below	104 (29.71)
Middle school or high school or equivalent	206 (58.86)
Junior college or above	40 (11.43)
**Marital status**	
Single	8 (2.29)
Married	327 (93.43)
Otherwise	15 (4.28)
**Having comorbidities**	
Yes	215(61.43)
No	135 (38.57)
**Having smoking history**	
Yes	93 (26.57)
No	257 (73.43)
**Medical burden**	
None	66 (18.86)
Mild	205 (58.57)
Moderate	78 (22.29)
Severe	1 (0.28)

### 3.2 Prevalence and score of symptoms

Table 2 displays the frequency and severity of each symptom using the Symptom Assessment Scale in patients with PCPF. The median number of reported symptoms was 7. The five most frequent symptoms were fatigue (77.71%), cough (72.86%), reduced sense of smell (68.57%), palpitations or rapid heart rate (61.43%), and shortness of breath (60.29%). The five most severe symptoms were fatigue (1.76 ± 1.10), cough (1.51 ± 1.13), reduced sense of smell (1.45 ± 1.17), palpitations or rapid heart rate (1.29 ± 1.11), and shortness of breath (1.22 ± 1.13).

**Table 2:** Prevalence and score of symptoms (*N* = 350).

**Variable of symptom**	**Prevalence (*n*, %)**	**Score (0–4) (Mean ±SD)**
Hyposmia	240 (68.57)	1.45 ± 1.17
Cough	255 (72.86)	1.51 ± 1.13
Dyspnea	211 (60.29)	1.22 ± 1.13
Chest pain	75 (21.43)	0.42 ± 0.85
Chest tightness	86 (24.57)	0.49 ± 0.91
Palpitations or Tachycardia	215 (61.43)	1.29 ± 1.11
Night sweats	190 (54.29)	1.10 ± 1.09
Chills	140 (40.00)	0.79 ± 1.03
Fatigue	272 (77.71)	1.76 ± 1.10
Arthralgia	148 (42.29)	0.83 ± 1.07
Myalgia	152 (43.43)	0.81 ± 1.01
Dizziness	72 (20.57)	0.41 ± 0.84
Headache	67 (19.14)	0.35 ± 0.76
Anxiety	89 (25.43)	0.55 ± 0.99
Depression	47 (13.43)	0.25 ± 0.67
Dysgeusia	125 (35.71)	0.65 ± 0.94
Diarrhea	53 (15.14)	0.28 ± 0.71
Nausea or vomiting	112 (32.00)	0.59 ± 0.94
Anorexia	135 (38.57)	0.74 ± 1.03

### 3.3 Prevalence and composition of symptom clusters

**Figure 1:**
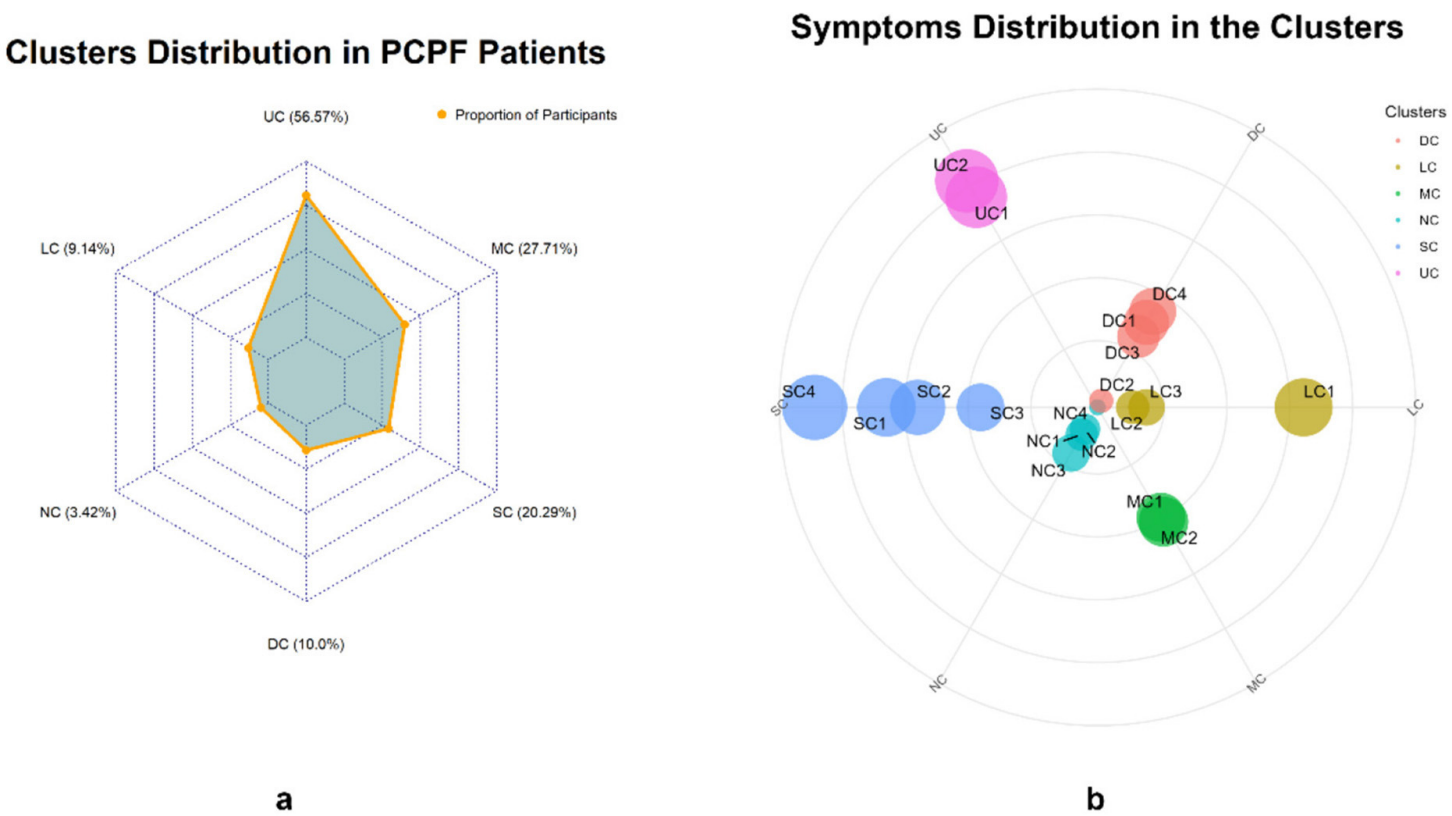
Distribution of symptom groups **(a)** and weight of internal symptoms **(b)**.

Table 3 presents the factor loadings for each symptom and the symptom clusters. The KMO (Kaiser-Meyer-Oklin inspections) value was 0.745, and Bartlett's test of sphericity yielded χ^2^ = 2,468.778, *P* < 0.001, indicating that the 19 symptom items in this study shared common factors and were suitable for factor analysis. Six common factors with eigenvalues >1 were extracted and classified into six symptom clusters: Upper Respiratory Tract Symptom Cluster (USC), Muscular and Joint Symptom Cluster (MSC), Somatic Symptom Cluster (SSC), Digestive Symptom Cluster (DSC), Lower Respiratory Tract Symptom Cluster (LSC), and Neurological and Psychological Symptom Cluster (NSC). The prevalence rates of these clusters were 56.57%, 27.71%, 20.29%, 10.00%, 9.14%, and 3.42%, respectively (Table 3 and Figure 1). Cronbach's alpha coefficients for the six symptom clusters were all >0.6, indicating acceptable internal consistency.

**Table 3:** Summary of symptom cluster.

**Cluster**	**Cluster composition**	**Factor loading**	**Number of participants (%)**	**Cronbach's Alpha**
Upper respiratory symptom	Hyposmia	0.843	198 (56.57)	0.640
	Cough	0.774		
Lower respiratory symptom	Dyspnea	0.608	32 (9.14)	0.600
	Chest pain	0.796		
	Chest tightness	0.791		
Somatic symptom	Palpitations or Tachycardia	0.735	71 (20.29)	0.672
	Night sweats	0.744		
	Chills	0.649		
	Fatigue	0.684		
Muscle and joint symptom	Arthralgia	0.820	97 (27.71)	0.603
	Myalgia	0.612		
Neuropsychological symptom	Dizziness	0.732	12 (3.42)	0.776
	Headache	0.623		
	Anxiety	0.876		
	Depression	0.680		
Digestive tract symptom	Dysgeusia	0.838	35 (10.00)	0.808
	Diarrhea	0.658		
	Nausea or vomiting	0.846		
	Anorexia	0.878		

### 3.4 Symptom networks and centrality indicators

**Figure 2:**
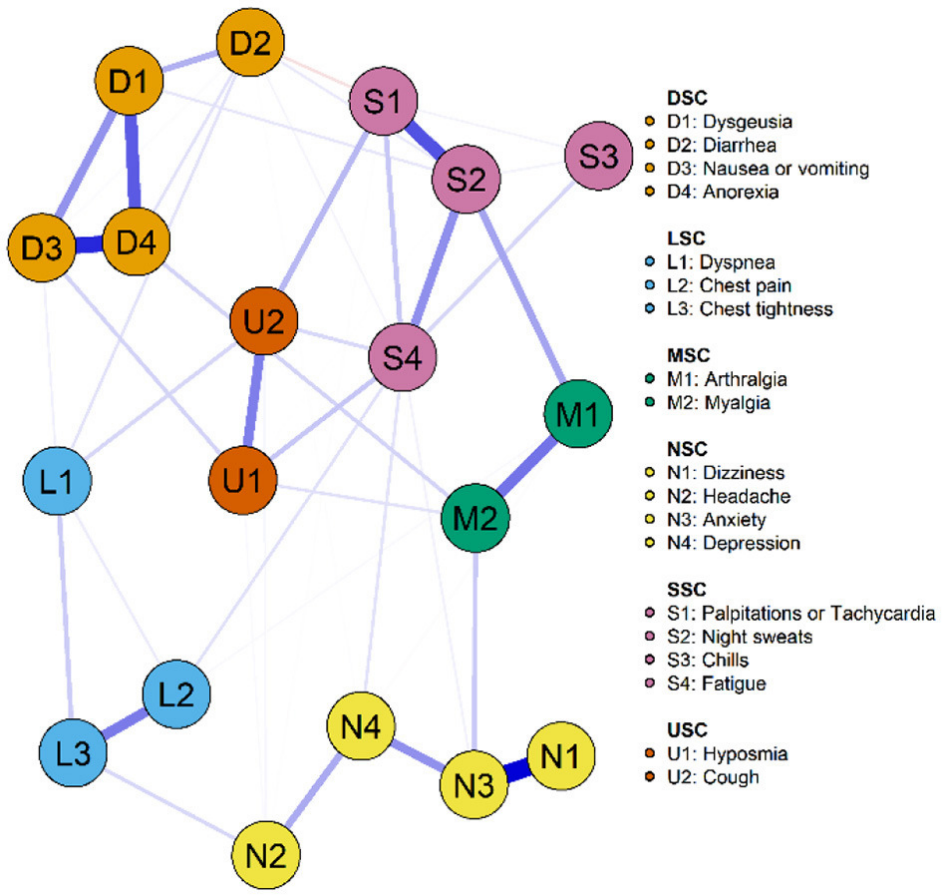
Symptom network of PCPF patients. “Nodes” represent symptoms, and “edges” represent the relationships between symptoms. Red edges indicate negative correlations, while blue edges indicate positive correlations. Thicker edges, closer distances, and darker colors signify stronger correlations between nodes, whereas thinner edges, farther distances, and lighter colors signify weaker correlations between nodes.

**Figure 3:**
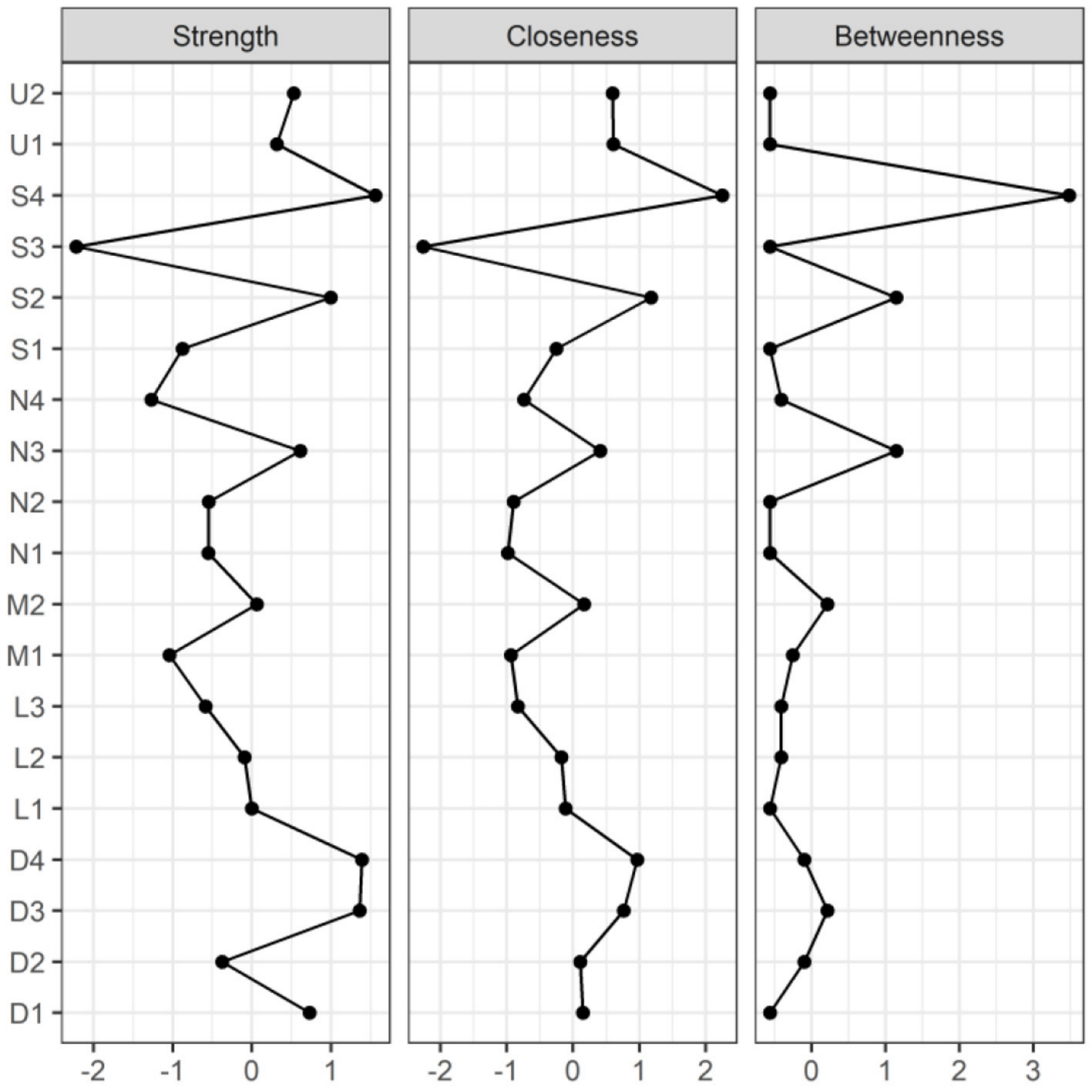
Centrality indicators of symptoms' network nodes. D1, Dysgeusia; D2, Diarrhea; D3, Nausea or vomiting; D4, Anorexia; L1, Dyspnea; L2, Chest pain; L3, Chest tightness; M1, Arthralgia; M2, Myalgia; N1, Dizziness; N2, Headache; N3, Anxiety; N4, Depression; S1, Palpitations or Tachycardia; S2, Night sweats; S3, Chills; S4, Fatigue; U1, Hyposmia; U2, Cough.

**Figure 4:**
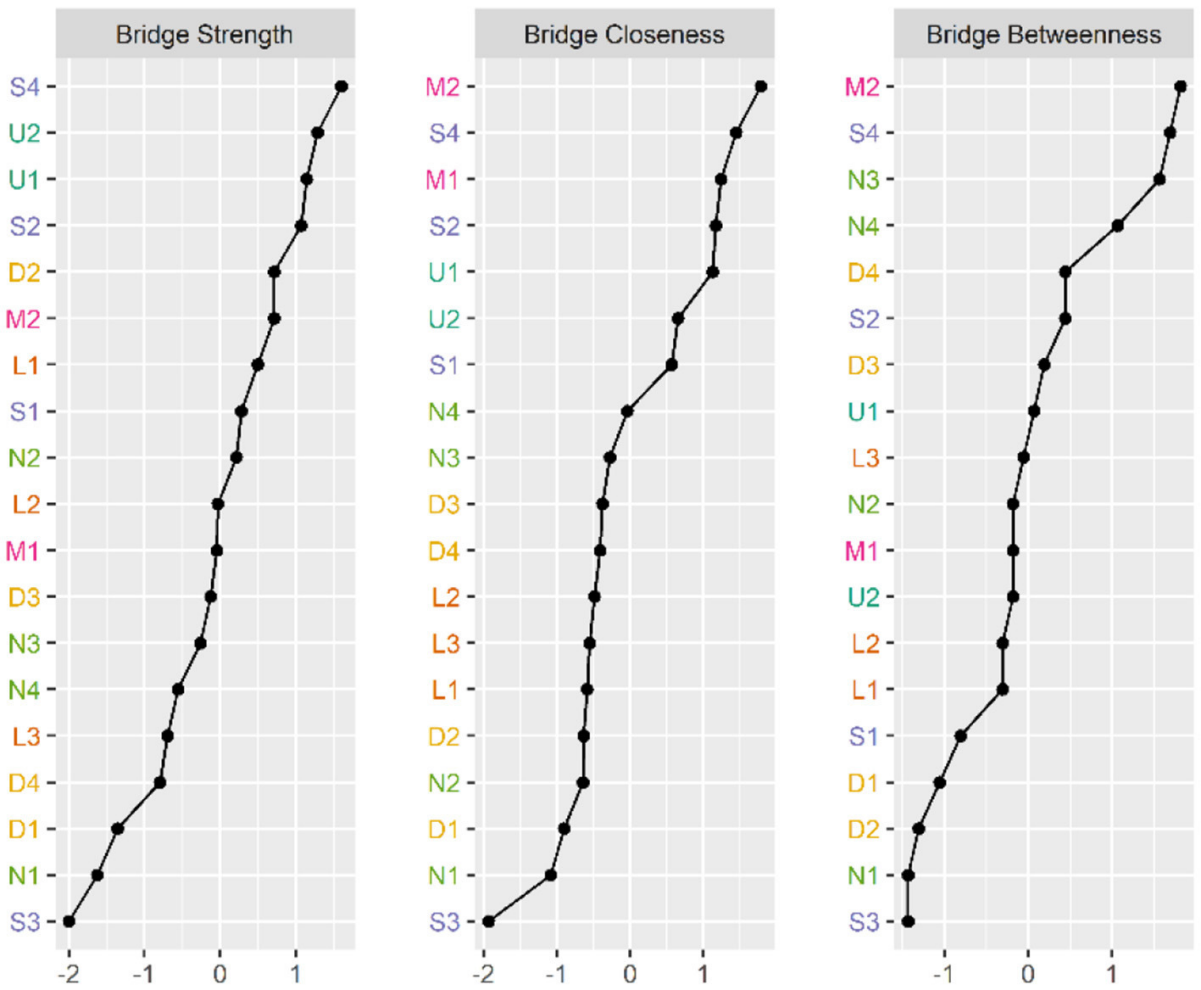
Bridge Centrality indicators of symptoms' network nodes. D1, Dysgeusia; D2, Diarrhea; D3, Nausea or vomiting; D4, Anorexia; L1, Dyspnea; L2, Chest pain; L3, Chest tightness; M1, Arthralgia; M2, Myalgia; N1, Dizziness; N2, Headache; N3, Anxiety; N4, Depression; S1, Palpitations or Tachycardia; S2, Night sweats; S3, Chills; S4, Fatigue; U1, Hyposmia; U2, Cough.

Figures 2, 3 illustrate the network associations and centrality indices of the 19 symptoms, respectively. Based on the thickness of the edges in the symptom network and the analysis results, the top three symptom pairs in terms of connection strength were “Anxiety” and “Dizziness” (r = 0.53), “Nausea or vomiting” and “Anorexia” (r = 0.42), and “Chest tightness” and “Chest tightness” (r = 0.34). The most central symptom in the symptom network was fatigue (r_S_ = 5.09, r_C_ = 0.01, r_B_ = 26), followed by loss of appetite (r_S_ = 4.96, r_C_ = 0.01, r_B_ = 3) and nausea or vomiting (r_S_ = 4.94, r_C_ = 0.01, r_B_ = 5). Figure 4 shows that fatigue had the highest bridge strength (r_Bs_ = 0.60, r_bC_ = 0.07, r_bB_ = 25), followed by cough (r_Bs_ = 0.55, r_bC_ = 0.07, r_bB_ = 10) and a reduced sense of smell (r_Bs_ = 0.53, r_bC_ = 0.07, r_bB_ = 12). Therefore, fatigue, cough, and a reduced sense of smell are the bridge symptoms in the symptom cluster network, connecting the USC and SSC.

### 3.5 Stability of symptom NA centrality indicators

**Figure 5:**
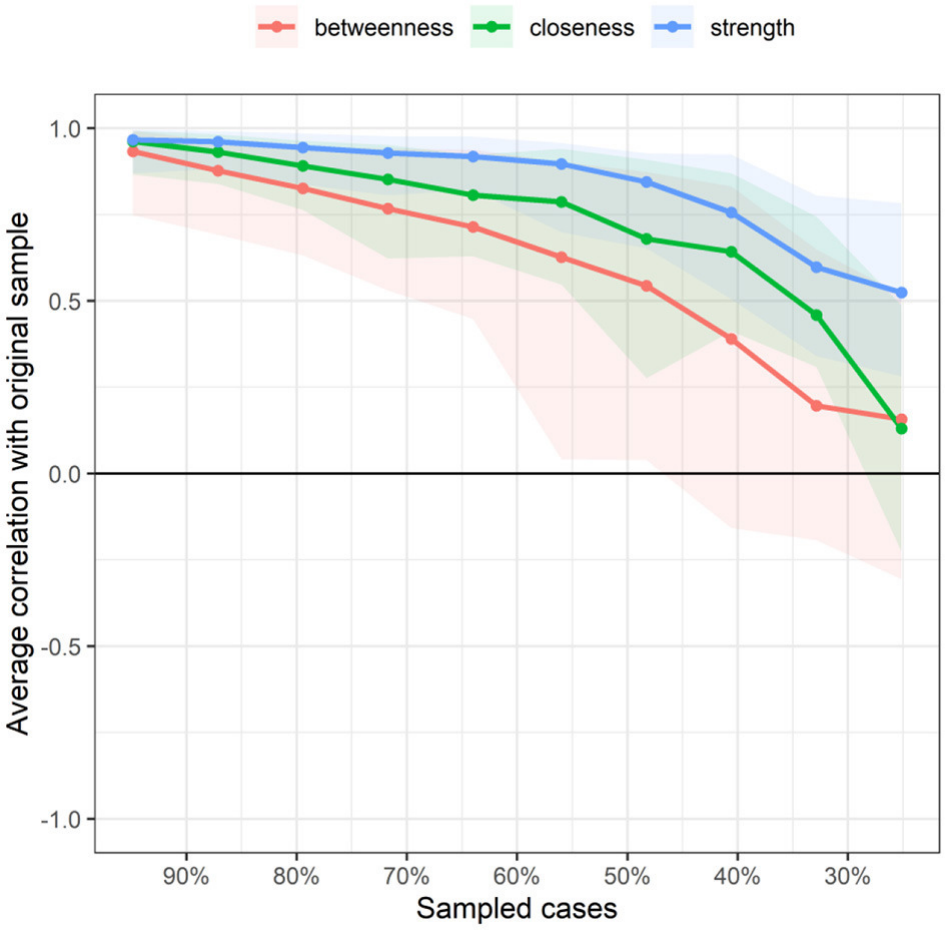
Related stability analysis of symptom network. The red area represents the accuracy of Betweenness, the green area represents the accuracy of Closeness, and the blue area represents the accuracy of Strength. The smaller the area, the smaller the 95% confidence interval, and the higher the accuracy of the centrality index.

Figure 5 displays the stability of centrality indices. As the sample size decreased, the stability of the betweenness centrality index and closeness centrality index significantly decreased, whereas the stability of the strength centrality index showed less variation and remained relatively high. The CS (Centrality Stability Coefficient and CS-coefficient) coefficient results indicated that the betweenness centrality coefficients was <0.25, demonstrating poor stability. The closeness centrality coefficient is 0.283. The strength centrality coefficient (CS[COR = 0.7] = 0.517) was > 0.50, indicating better stability.

### 3.6 Symptom cluster networks and centrality indicators

**Figure 6:**
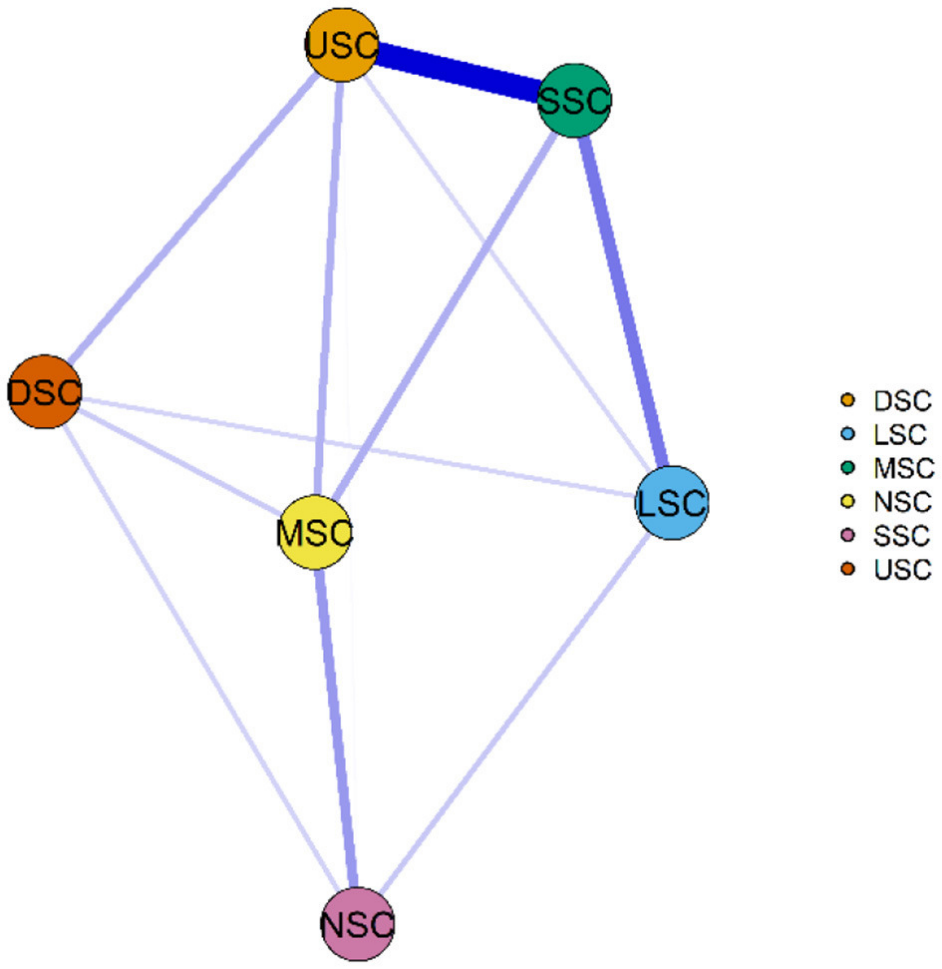
Symptom cluster network of PCPF patients. “Nodes” represent symptom Clusters, and “edges” represent the relationships between symptoms. Red edges indicate negative correlations, while blue edges indicate positive correlations. Thicker edges, closer distances, and darker colors signify stronger correlations between nodes, whereas thinner edges, farther distances, and lighter colors signify weaker correlations between nodes. USC, Upper Respiratory Tract Symptom Cluster; MSC, Muscular and Joint Symptom Cluster; SSC, Somatic Symptom Cluster; DSC, Digestive Symptom Cluster; LSC, Lower Respiratory Tract Symptom Cluster; NSC, Neurological and Psychological Symptom Cluster.

**Figure 7:**
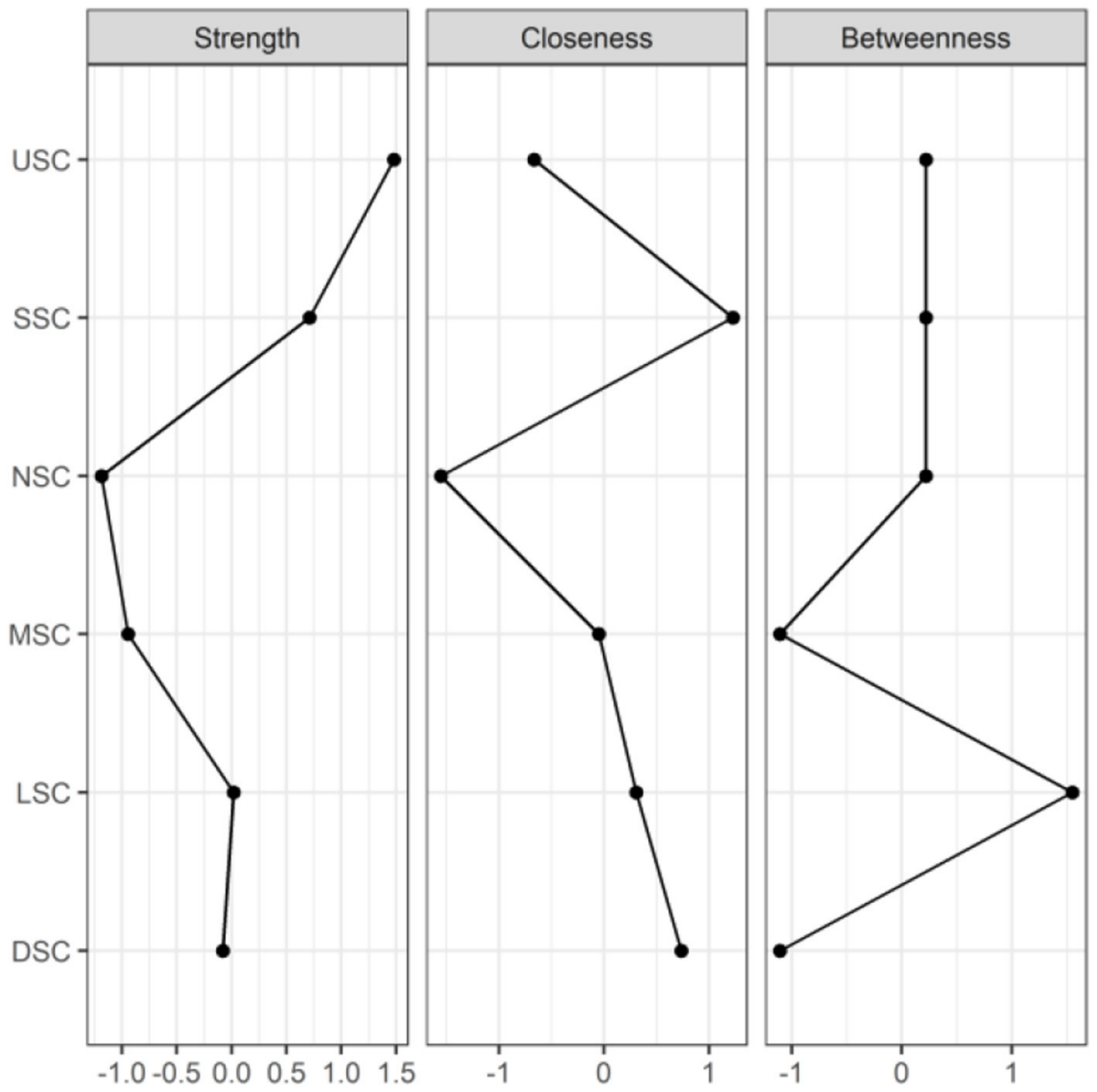
Centrality indicators of symptoms' network nodes. USC, Upper Respiratory Tract Symptom Cluster; MSC, Muscular and Joint Symptom Cluster; SSC, Somatic Symptom Cluster; DSC, Digestive Symptom Cluster; LSC, Lower Respiratory Tract Symptom Cluster; NSC, Neurological and Psychological Symptom Cluster.

**Figure 8:**
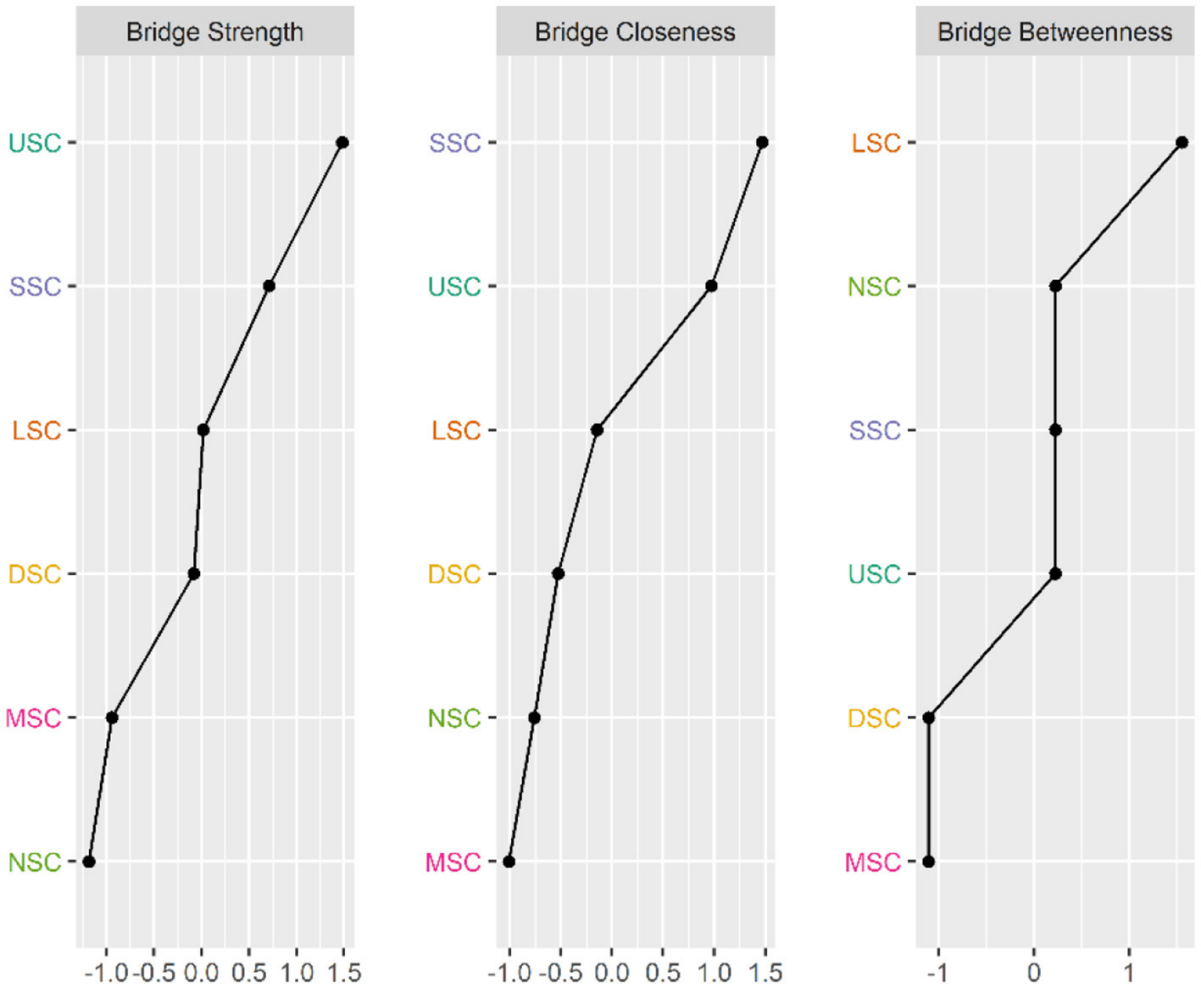
Bridge Centrality indicators of symptoms cluster' network nodes. USC, Upper Respiratory Tract Symptom Cluster; MSC, Muscular and Joint Symptom Cluster; SSC: Somatic Symptom Cluster; DSC, Digestive Symptom Cluster; LSC, Lower Respiratory Tract Symptom Cluster; NSC, Neurological and Psychological Symptom Cluster.

Figures 6, 7 illustrate the network associations and centrality indices of the six symptom clusters, respectively. The top three edges in terms of connection strength were between “USC“ and ”SSC“ (r = 0.48), ”USC“ and ”DSC“ (r = 0.36), and ”LSC“ and ”NSC“ (r = 0.36). In the symptom cluster network, ”USC (r_S_ = 1.66, r_C_ = 0.06, r_B_ = 0)” was the most central symptom cluster, followed by “SSC” (r_S_ = 1.59, r_C_ = 0.06, r_B_ = 0). Figure 8 shows that USC had the highest bridge strength (r_Bs_ = 0.81, r_bC_ = 0.18, r_bB_ = 1), followed by SSC (r_Bs_ = 0.74, r_bC_ = 0.18, r_bB_ = 1). Consequently, USC and SSC are bridge symptom clusters in the symptom cluster network.

### 3.7 Stability of symptom cluster NA centrality indicators

**Figure 9:**
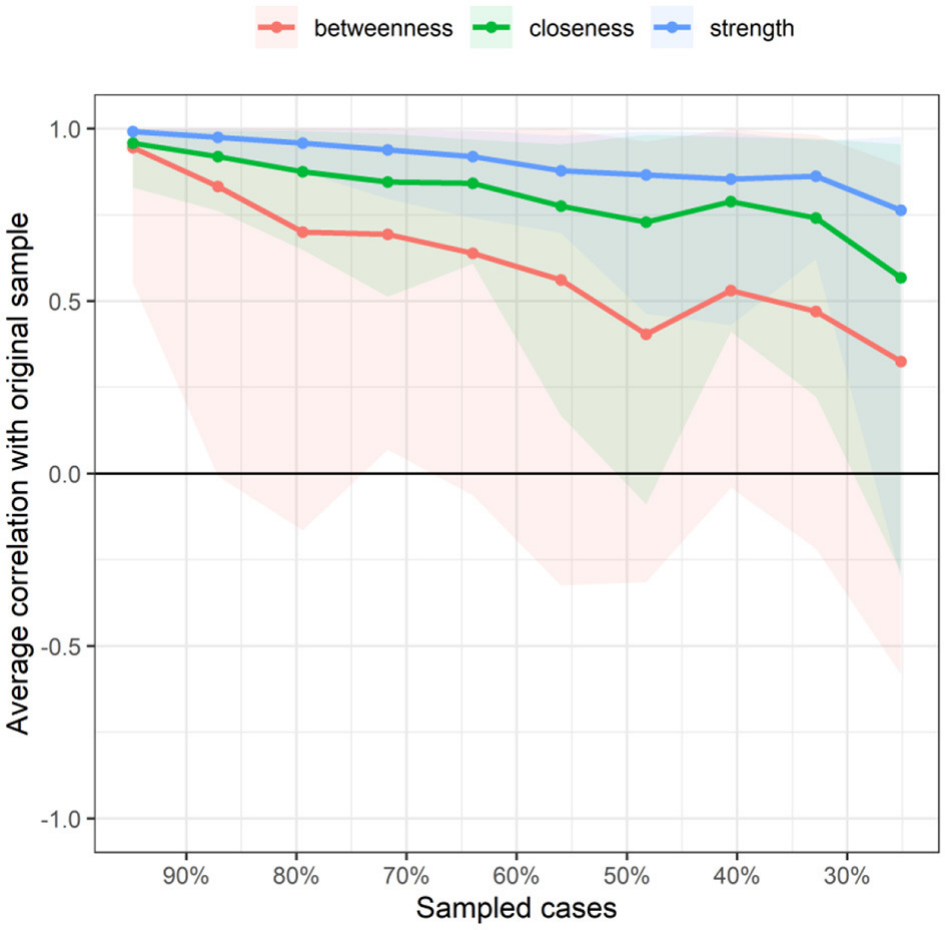
Related stability analysis of symptom cluster network. The red area represents the accuracy of Betweenness, the green area represents the accuracy of Closeness, and the blue area represents the accuracy of Strength. The smaller the area, the smaller the 95% confidence interval, and the higher the accuracy of the centrality index.

Figure 9 indicates the stability of centrality indices. As the sample size decreased, the stability of the betweenness centrality index and closeness centrality index significantly decreased, whereas the stability of the strength centrality index showed less variation and remained relatively high. The CS coefficient results indicated that both the betweenness and closeness centrality coefficients were <0.25, demonstrating poor stability. The strength centrality coefficient (CS[COR = 0.7] = 0.517) was > 0.50, indicating better stability.

### 3.8 Multiple linear regression models for overall symptom scores and symptom clusters

Table 4 presents the results of the multiple linear regression models for the overall symptom scores and scores of each symptom cluster dimension in patients with PCPF. Female patients (*P* < 0.001), older age (*P* < 0.001), higher education level (*P* < 0.01), history of smoking (*P* < 0.001), being one's own primary caregiver (*P* < 0.01), and comorbidities (*P* < 0.001) were associated with higher overall symptom scores. Variables such as gender, age, educational level, smoking history, and primary caregiver status were associated with the severity of the six symptom clusters.

**Table 4:** Multiple linear regression of six symptom clusters (*N* = 350).

**Variable**	**Model 1 Overall**	**Model 2** **Upper respiratory symptom**	**Model 3 Lower respiratory symptom**	**Model 4** **Somatic symptom**	**Model 5 Muscle and joint symptom**	**Model 6** **Neuropsychological symptom**	**Mode 7 Digestive tract symptom**
Male (compared to female)	0.279^c^	0.228 ^c^	0.208 ^c^	0.165^b^	0.239^c^	0.115^a^	0.160^b^
age	0.245 ^c^	0.129^b^	0.156^b^	0.210 ^c^	0.164^b^	0.048	0.180 ^c^
Middle school or high school (compared to Primary school or less)	−0.124^b^	−0.114 ^a^	−0.047	0.148^b^	0.029	0.060	0.060
Living in urban (compared to living in rural)	0.048	0.065	0.024	0.020	0.037	0.046	0.020
Smoking (compared to no smoking)	−0.215^c^	−0.149^b^	−0.236^c^	−0.106^a^	−0.129^a^	−0.054^a^	−0.122^a^
Employed (compared to otherwise)	0.002	−0.017	0.017	−0.003	−0.101	−0.014	0.033
Marital status: Married (compared to otherwise)	−0.002	−0.028	0.038	0.006	−0.035	−0.054	0.029
Primary caregiver during treatment: myself (compared to otherwise)	−0.116^b^	−0.094	−0.146^b^	−0.037	−0.018	−0.200^c^	−0.044
Having no comorbidity (compared to having comorbidity)	0.274^c^	0.303^c^	0.127^a^	0.260^c^	0.085	0.090	0.156^b^

Model 1: *F* = 18.694, *P* < 0.001, $R_{\text{adj}}^{2}$ = 0.313; Model 2: *F* = 11.982, *P* < 0.001, $R_{\text{adj}}^{2}$ = 0.221; Model 3: *F* = 8.470, *P* < 0.001, $R_{\text{adj}}^{2}$ = 0.162; Model 4: *F* = 9.531, *P* < 0.001, $R_{\text{adj}}^{2}$ = 0.180; Model 5: *F* = 5.029, *P* < 0.001, $R_{\text{adj}}^{2}$ = 0.094; Model 6: *F* = 3.963, *P* < 0.001, $R_{\text{adj}}^{2}$ = 0.071; Model 7: *F* = 5.314, *P* < 0.001, $R_{\text{adj}}^{2}$ = 0.100. ^a^*P* < 0.05. ^b^*P* < 0.01. ^c^*P* < 0.001.

## 4 Discussion

In this study, network analysis was used for the first time to identify the symptom network of patients with PCPF. Compared with the previous symptom clustering in Long COVID study (51), the main contribution of our study is to provide a more comprehensive and in-depth understanding of the core symptom clusters in PCPF patients, and to analyze the demographic and disease-related factors associated with these symptom clusters. Based on network analysis, our analysis provides valuable information for policy makers and medical experts by studying the core symptom clusters in PCPF patients and the relationships between symptoms in detail, to promote precise symptom management and improve the efficiency of symptom management.

Six symptom clusters were identified, namely, USC, LSC, SSC, NSC, DSC, and MSC. In the overall symptom network, USC was the most central cluster based on three centrality indices (strength, closeness, and betweenness). Gender, age, educational level, smoking history, and primary caregiver status were associated with the severity scores of the six symptom clusters.

The first symptom cluster was USC, which is the most common symptom cluster. Upper respiratory symptoms include a reduced sense of smell and cough. Unlike previous studies (34), which uniformly categorized nasal mucus, sore throat, sputum, and cough as respiratory symptoms, our study identified them separately. This difference could be due to the fact that prior studies included patients with acute-phase COVID-19 who experienced severe nasal symptoms (34). In contrast, our study included patients with PCPF who developed pulmonary fibrosis after COVID-19, typically 3 weeks post-infection, when nasal symptoms significantly decreased. Cough may reflect virus-related bronchial hyperreactivity or chronic bronchitis (29), whereas a reduced sense of smell could be closely related to damage to olfactory epithelial cells caused by the coronavirus D614G mutation of the coronavirus (52, 53).

Additionally, we found that the USC had the highest values for the strength and bridge centrality indices, indicating that it was the core and bridge symptom cluster in the symptom network. Core symptoms can diffuse intervention effects to the peripheral nodes, ultimately leading to the alleviation or disappearance of other symptoms (54). Identifying bridge symptoms as the focus of clinical symptom management interventions can disrupt connections between symptom clusters, deactivate related symptom clusters, and improve the efficiency and precision of clinical interventions (54). In the present study, USC was associated with SSC, LSC, and DSC. Interventions in the USC can alleviate the symptoms of SSC, LSC, and DSC. Thus, the evaluation and treatment of upper respiratory tract symptom cluster should be considered a crucial component of PCPF care and support, prioritizing interventions targeting upper respiratory symptom clusters to minimize patient symptom burden.

In our study, cough was the most prevalent symptom in this cluster (72.9%), and frequent coughing can cause severe fatigue, chest pain, shortness of breath, oxygen desaturation, and anxiety (55). Studies (56) suggest that persistent cough induced by SARS-CoV-2, if not addressed, may eventually lead to asthma. Currently, there are no specific management guidelines for cough related to PCPF, and general guidelines for chronic cough management can be followed (55). Neuromodulators may be considered in the treatment of persistent cough in patients with PCPF. However, for patients with long-term COVID-19 who have normal imaging findings, the use of neuromodulators should be cautious as they can exacerbate neurological symptoms such as fatigue and dizziness (55). Clinical trials are currently underway to evaluate the efficacy of nintedanib and pirfenidone for PCPF (55). Olfactory dysfunction significantly affects quality of life and is associated with depression (57, 58). Some studies (58) suggest that combining systemic corticosteroids, glycosaminoglycan-based antithrombotic drugs (mesoglycan), and diuretics may effectively treat olfactory and taste disorders that appear 30 days after COVID-19 resolution. Nasal immunotherapy with soluble factors from M2 macrophages has the potential to alleviate olfactory dysfunction in patients with Long COVID-19 (59). Notably, even in patients with pulmonary fibrosis, these patients primarily report upper respiratory symptoms. Nurses should focus on these patients and increase the symptom assessment frequency to prevent further lung fibrosis and severe lower respiratory symptoms.

The second symptom cluster was LSC, which included chest pain, chest tightness, and shortness of breath. Unlike previous studies (39) that did not differentiate between upper and lower respiratory symptoms, our principal component analysis did. This difference may be because our study did not distinguish between the types of coronavirus strains in the patients' initial infections (39, 60, 61). Different strains can cause significantly different symptoms (60, 61). Shortness of breath and chest tightness reflect the impact of pulmonary fibrosis on oxygenation, whereas chest pain indicates cardiovascular damage caused by coronavirus (62, 63). LSC can lead to anxiety and affect quality of life (64). Healthcare providers should promptly assess this symptom cluster and offer respiratory rehabilitation training, positional management, psychological support, and continuous monitoring of changes in patients' conditions (64).

The third symptom cluster was SSC, which included fatigue, increased heart rate or palpitations, night sweats, and chills. Our study found that fatigue was the most common symptom (77.7%), consistent with many studies (28–31, 38, 65–67). For instance, Cornelissen et al. found that 75.9% of patients reported fatigue within 3–6 months post-COVID-19 infection, and 57.1% reported fatigue within 9–12 months (67). In the symptom network, fatigue had the highest values for the strength and bridge centrality indices, indicating that fatigue was the core and bridge symptom in the network. Intervention in fatigue can alleviate multiple systemic symptoms in patients with PCPF, making it a precise target for symptom management.

Fatigue is not only a hallmark of SSC but also a common manifestation of PCPF, representing the interactions between different systems. Fatigue, palpitations, night sweats, and chills forming a symptom cluster may reflect systemic inflammation and autonomic nerve damage in post-COVID-19 patients (68–70). Despite its significance, fatigue as a post-COVID-19 sequela has not yet received sufficient attention. It can be assessed through self-reports and direct evaluations of symptom perception and declines in physical and cognitive performance. Regular follow-up assessments of fatigue symptoms in patients with PCPF are crucial for improving their quality of life (71). Comprehensive diagnostic and personalized treatment strategies, multidisciplinary teams, and encouraging patient involvement in symptom management can benefit fatigue alleviation (72). Non-pharmacological interventions, such as pacing and highly individualized outpatient care, are recommended for nurses to improve symptom burden in patients with PCPF (73–75).

The fourth symptom cluster is MSC, which comprises muscle and joint pain. A systematic review of post-COVID-19 pain reported high frequencies of muscle and joint pain and found that nearly 10% of SARS-CoV-2 infections resulted in musculoskeletal symptoms within the 1^st^ year post-infection (76). Fluctuations in pain intensity and frequency reflect the recurrent and remissive nature of post-COVID-19 conditions. Several studies (30, 38) have defined joint pain and muscle pain as musculoskeletal symptoms that affect the quality of life. However, some studies (27, 31) have combined myalgia with fatigue, viewing it as part of myalgic encephalomyelitis/chronic fatigue syndrome (77), a complex multisystem disease resulting from viral, bacterial, or parasitic infections characterized by muscle and joint pain, fatigue, cognitive impairment, orthostatic intolerance, and sleep disorders. It is associated with neurological damage, immune dysfunction, redox imbalance, and defects in energy metabolism (77, 78). The symptom network in our study showed connections between MSC, SSC, and NSC, echoing these insights. Musculoskeletal pain can limit physical function and is closely associated with psychological issues, such as depression and anxiety (79). Therefore, healthcare providers should actively assess pain levels in patients with PCPF and employ both pharmacological and non-pharmacological therapies to alleviate pain and improve the quality of life.

The fifth symptom cluster is NSC, which includes dizziness, headache, anxiety, and depression. Previous studies have reported the prevalence of these symptoms (28–30, 37), which is consistent with our findings. Analgesics, psychotherapy, and anti-anxiety and antidepressant medications are effective in patients with this symptom cluster (37). Clinical nurses should provide health education to promote treatment adherence and monitor adverse reactions to medications.

The sixth symptom cluster is DSC, which includes a reduced sense of taste, diarrhea, loss of appetite, and nausea or vomiting. The mechanisms underlying the gastrointestinal symptoms of COVID-19 are not well understood. Direct viral cytopathic effects in the mucosa and indirect effects of systemic cytokine storms can lead to gastrointestinal symptoms (80). Existing evidence highlights the prevalence of gastrointestinal symptoms post-COVID-19 (81). A study from the United States using exploratory factor analysis investigated symptom clusters in 999 patients 1 year post-COVID-19 infection and found prevalence rates of 23.7% for diarrhea, 22.5% for abdominal pain, and 15.2% for nausea/vomiting, indicating lower rates compared to other systems' symptoms (38). The importance of gastrointestinal symptoms remains unclear despite numerous studies on post-COVID-19 symptoms. Several factors have contributed to this ambiguity. Most COVID-19 studies are retrospective, and the importance of gastrointestinal symptoms may be underreported or unrecorded compared to severe respiratory and somatic symptoms. Additionally, nausea and diarrhea are often subjectively assessed, making their definitions prone to misunderstanding. Certain antibiotics, antiviral drugs, and enteral.

In our study, the gastrointestinal symptom cluster was connected to several symptom clusters, such as the upper respiratory, lower respiratory, muscle and joint, and neuropsychological symptom clusters, suggesting a complex connection of the gastrointestinal symptom cluster in the symptom network. Gastrointestinal symptoms can affect the nutritional intake of patients, leading to malnutrition, weight loss, and even decreased immunity, thereby prolonging the duration of other symptom clusters (82, 83). The management of gastrointestinal symptoms should be based on individual symptoms and potential conditions, combined with multidisciplinary teams to provide symptomatic and specific treatment, drug and non-drug intervention, diet adjustment, supportive care, complication management, and follow-up care (84). For clinical nurses, early identification and timely management of gastrointestinal symptom clusters and the treatment of complications can improve the prognosis of patients (85).

Consistent with previous studies (27, 86), we found that women had higher comprehensive symptom scores and more severe symptom clusters than men. There are two possible reasons for this. First, women have a lighter response to viral infections and a higher antibody response. Early infection is conducive to clearing the virus, but the incidence of adverse reactions to vaccines and antiviral drugs is also higher. Second, X-linked genes are considered to affect susceptibility to viral infections and autoimmune diseases, provide support for the autoimmune process, and thus play a role in the development of the novel Long COVID-19 pneumonia. Based on these two points, female patients have mild symptoms in the acute phase and severe symptoms in the later stage (87). However, the specific mechanism behind this gender difference leading to different severities of symptoms is still under study.

We found that age was positively correlated with the severity of the upper respiratory symptom clusters. Contrary to the results of the study (34, 39), two studies showed that age was negatively correlated with the severity of olfactory and gustatory disorders, suggesting that it may be related to the decline of immunity and degradation of self-repair function in the elderly (88, 89). Reduced naive CD8 T cell abundance and expression of antiviral defense genes (IFITM3 and TRIM22) have been found in elderly patients with severe COVID-19 (89). The subjects included in these two studies were mostly in the acute phase. During this period, the immune response of the elderly was weak, and the inflammatory response was light. Additionally, the olfactory and taste sensations in elderly patients in the acute phase decreased, resulting in no obvious olfactory and taste disorders. The patients in this study had PCPF and were basically not in the acute phase. Elderly patients had prolonged inflammation and persistent viruses due to insufficient immune response in the early stage of viral infection, which causes extensive tissue damage in various organs and systems, resulting in severe olfactory and taste disorders (33). Furthermore, age was positively correlated with the other five symptom clusters and the comprehensive score of symptoms, consistent with the general consensus in the current literature, and a possible reason was also related to the above mechanism. The current study found that higher education levels were associated with more severe upper respiratory and somatic symptom clusters. This may be because the education level of respondents might affect self-reported data; that is, people with high education levels are usually better at accurately reporting and describing health problems (90). In accordance with a previous study (91), those with a history of smoking had higher comprehensive symptom scores and more severe symptom clusters. This may be because smoking can damage the olfactory neuroepithelial system, leading to olfactory dysfunction or loss (92). Additionally, smoking not only leads to respiratory diseases but also affects the human cardiovascular system, nervous system, digestive system, and other multisystem diseases, making various symptom clusters more serious (93, 94). This study also found that patients who themselves were primary caregivers had higher comprehensive symptom scores and more severe lower respiratory and neuropsychological symptom clusters. A possible reason may be that patients need to bear the dual burden of daily life and disease management alone and cannot focus on managing and relieving symptoms. We also found that patients with comorbidities were more likely to exhibit severe systemic symptoms. Our results are similar to previous studies (95, 96) that reported that comorbidities were associated with the severity and duration of symptoms in the long novel coronary. Therefore, healthcare professionals must focus on patients with PCPF, particularly females, the elderly, highly educated, those with a history of smoking, or those with high comorbidity in their clinical practice. Additionally, there is a need to actively assess the symptom clusters of such high-risk patients to improve their disease prognosis and quality of life.

## 5 Strengths and limitations

The strengths of our study lie in being the first to include patients with PCPF as the study population and employing network analysis to identify core symptom clusters. The identification of these symptom clusters adds new evidence to the clinical subtypes of PCPF and provides theoretical support for future precise symptom interventions for patients with PCPF. However, this study has some limitations. First, our analysis only encompassed 19 symptoms, neglecting many others. Future studies should aim to investigate a broader range of symptoms and establish the core symptom and symptom cluster more comprehensively in a large-scale, multi-center study. Second, our study is a contemporaneous symptom network constructed from a cross-sectional dataset obtained through convenience sampling, which limits the generalizability and external validity of the study results and does not allow for the verification of causal relationships between symptoms and symptom clusters. Therefore, it is necessary to conduct longitudinal studies to explore dynamic networks and to identify symptoms and symptom clusters that adversely affect others. Third, multivariate linear models assume a linear relationship between the independent and dependent variables, but in reality there may be non-linear correlations, and the goodness of fit of the model (e.g., R^2^) may be underestimated, leading to misinterpretation of the direction of the effect. Fourth, the study does not take into account when the individuals contracted COVID-19 and the time gap after which they developed fibrosis, which may impact the reliability of our findings. Additionally, this study did not distinguish between the different strains of coronavirus leading to pulmonary fibrosis, which limits the generalizability of the findings across different strains.

## 6 Implications for research and clinical practice

The main finding of this study was the identification of six symptom clusters (USC, LSC, SSC, musculoskeletal symptom cluster, neuropsychological symptom cluster, and gastrointestinal symptom cluster), which are significant for PCPF research and clinical practice. Identification of these symptom clusters adds new evidence to the clinical subtypes of patients with PCPF and highlights the central role of the upper respiratory tract symptom cluster. Identifying symptom clusters in PCPF is an important step toward a better understanding of the condition of patients with PCPF and its underlying mechanisms. Network analysis of symptom clusters can help suggest one or more etiologies and pathological mechanisms of PCPF, providing a reference for evidence-based treatment and care.

In clinical practice, this underscores the necessity for precise interventions for upper gastrointestinal symptom clusters and fatigue symptoms. Additionally, when developing intervention measures, patient-specific characteristics should be considered, with a focus on evaluating patients who are female, older, have higher educational levels, have a smoking history, or are primary caregivers to improve disease prognosis and quality of life.

## 7 Conclusion

This study primarily used network analysis to identify six symptom clusters in patients with PCPF: USC, LSC, SSC, musculoskeletal symptom cluster, neuropsychological symptom cluster, and gastrointestinal symptom cluster. Fatigue had the highest incidence and was identified as a core and bridge symptom. Similarly, USC was recognized as both a core symptom cluster and a bridge symptom cluster. Prioritizing interventions targeting fatigue and USC is crucial for effective symptom management in patients with PCPF.

## Data Availability

The raw data supporting the conclusions of this article will be made available by the authors, without undue reservation.
